# Patient and Clinician Characteristics Associated With Secure Message Content: Retrospective Cohort Study

**DOI:** 10.2196/26650

**Published:** 2021-08-19

**Authors:** Dawn Heisey-Grove, Cheryl Rathert, Laura E McClelland, Kevin Jackson, Jonathan P DeShazo

**Affiliations:** 1 The MITRE Corporation McLean, VA United States; 2 Department of Health Management and Policy College for Public Health and Social Justice Saint Louis University St. Louis, MO United States; 3 Department of Health Administration College of Health Professions Virginia Commonwealth University Richmond, VA United States; 4 Health Services Management Department of Nursing and Allied Health Norfolk State University Norfolk, VA United States

**Keywords:** patient-provider communication, electronic messaging, hypertension, diabetes

## Abstract

**Background:**

Good communication has been shown to affect patient outcomes; however, the effect varies according to patient and clinician characteristics. To date, no research has explored the differences in the content of secure messages based on these characteristics.

**Objective:**

This study aims to explore characteristics of patients and clinic staff associated with the content exchanged in secure messages.

**Methods:**

We coded 18,309 messages that were part of threads initiated by 1031 patients with hypertension, diabetes, or both conditions, in communication with 711 staff members. We conducted four sets of analyses to identify associations between patient characteristics and the types of messages they sent, staff characteristics and the types of messages they sent, staff characteristics and the types of messages patients sent to them, and patient characteristics and the types of messages they received from staff. Logistic regression was used to estimate the strength of the associations.

**Results:**

We found that younger patients had reduced odds of sharing clinical updates (odds ratio [OR] 0.77, 95% CI 0.65-0.91) and requesting prescription refills (OR 0.77, 95% CI 0.65-0.90). Women had reduced odds of self-reporting biometrics (OR 0.78, 95% CI 0.62-0.98) but greater odds of responding to a clinician (OR 1.20, 95% CI 1.02-1.42) and seeking medical guidance (OR 1.19, 95% CI 1.01-1.40). Compared with White patients, Black patients had greater odds of requesting preventive care (OR 2.68, 95% CI 1.30-5.51) but reduced odds of requesting a new or changed prescription (OR 0.72, 95% CI 0.53-0.98) or laboratory or other diagnostic procedures (OR 0.66, 95% CI 0.46-0.95). Staff had lower odds of sharing medical guidance with younger patients (OR 0.83, 95% CI 0.69-1.00) and uninsured patients (OR 0.21, 95% CI 0.06-0.73) but had greater odds of sharing medical guidance with patients with public payers (OR 2.03, 95% CI 1.26-3.25) compared with patients with private payers. Staff had reduced odds of confirming to women that their requests were fulfilled (OR 0.82, 95% CI 0.69-0.98). Compared with physicians, nurse practitioners had greater odds of sharing medical guidance with patients (OR 2.74, 95% CI 1.12-6.68) and receiving prescription refill requests (OR 3.39, 95% CI 1.49-7.71). Registered nurses had greater odds of deferred information sharing (OR 1.61, 95% CI 1.04-2.49) and receiving responses to messages (OR 3.93, 95% CI 2.18-7.11) than physicians.

**Conclusions:**

The differences we found in content use based on patient characteristics could lead to the exacerbation of health disparities when content is associated with health outcomes. Disparities in the content of secure messages could exacerbate disparities in patient outcomes, such as satisfaction, trust in the system, self-care, and health outcomes. Staff and administrators should evaluate how secure messaging is used to ensure that disparities in care are not perpetuated via this communication modality.

## Introduction

### Background

Appropriate use of health information technology may promote patient engagement and empowerment by improving patients’ preparation for, and recall of, clinical encounters [1]. One form of health information technology is secure messaging—the electronic exchange of messages between patients and clinicians, typically via a secure platform such as a patient portal. Published research highlights the potential of secure messaging to support patient satisfaction, access to care, and health outcomes. Most research has explored health care utilization, with a number of studies identifying reductions in patients’ visits associated with secure messaging [2-4]. Other studies have identified improvements in selected measures for screening and testing associated with secure messaging use [5-7]. Secure message use has also been associated with improvements in blood pressure control [5,8,9], glycemic levels [5-7,10-12], and improved postdischarge coping [13].

However, secure messaging use is associated with a variety of clinician and patient characteristics [14-16]. Furthermore, moderators between communication and patient health outcomes include both patient and clinician characteristics (eg, age, gender, race, income, and education) [17]. Consistent with this, research indicates that differential use by patients’ race and ethnicity persists once patients access patient portals [15]. If secure messaging is associated with improvements in patients’ satisfaction, access to care, and health outcomes but use varies according to patient and clinician characteristics, there is a chance that the benefits of secure messaging communication may be inequitably applied across populations, leaving some patients without the benefits of that form of communication.

Communication functions such as information exchange, emotional support, uncertainty management, and support for decision-making and self-management can be provided by clinicians through secure messaging, leading to changes in patients’ health outcomes [18]. Research has demonstrated associations between patients’ improved glycemic levels and diastolic blood pressure and clinicians’ information-sharing message content [12]. The same study found that negative message content (eg, denying patients’ requests and responses deferring answers to a later time) was associated with increased systolic blood pressure. Although published work demonstrates that communication strategies vary according to age, sex, race, primary language, and comfort level with the communication medium [19-23], the authors could find no research on whether differences in message content exist based on patient or clinician characteristics.

### Objectives

In this study, we explore whether differences exist in communication functions based on characteristics of patients and clinicians, representing the senders and receivers in secure message threads. Using a taxonomy created specifically for secure messages, we coded the patient- and staff-generated messages in a large sample of patient-initiated message threads [24]. We then explored the differences in message content prevalence based on the characteristics of senders and receivers. Our hypotheses for this research are as follows:

- Hypothesis 1 (patients as senders): message content sent by patients to staff will vary based on patients’ age, sex, race, health status, insurance type, and proximity to the clinic.
- Hypothesis 2 (staff as receivers): patients will vary their message content based on the staff type and clinical specialty of the intended recipient.
- Hypothesis 3 (staff as senders): message content in staffs’ replies will vary based on staff type, clinical specialty, and annual message volume.
- Hypothesis 4 (patients as receivers): staff will vary their message content based on patients’ age, sex, race, health status, and insurance type.

## Methods

### Study Population

Our study included adult patients with diabetes, hypertension, or both conditions selected from patients of a large urban medical center who sent secure messages using the outpatient portal of the medical center (Cerner) between January 1 and December 31, 2017. To ensure that patients were patients at the medical center for the study duration and their diagnoses persisted during that period, we included patients with relevant diagnosis codes in the years preceding (2016) and following (2018) the study period. Patients had to have at least two outpatient visits or 1 inpatient visit in 2016 with diagnosis codes for either diabetes (*International Classification of Diseases, Tenth Revision, Clinical Modification* [*ICD-10-CM*] E11) or hypertension (*ICD-10-CM* I10), and at least one outpatient visit between January and June 2018. We only included visits within the medical center.

We stratified patients who met the inclusion criteria based on their health condition (hypertension only, diabetes only, or both conditions). The required sample size necessitated the inclusion of all patients with diabetes. We used version 9.2 of the SAS System for Windows (SAS Institute Inc) to select a simple random sample from each of the other two strata. We then included all staff who were the intended recipients of, or who responded to, our sampled patients’ secure messages during the study period.

Our analyses included all threads initiated by the sampled patients, completed, and saved to patients’ charts between January 1 and December 31, 2017. Secure messages were extracted during a chart review of each patient’s electronic medical record. We did not include communications outside secure messaging in these analyses. This research was approved by the institutional review board.

Table S1 of Multimedia Appendix 1 includes the number and percentage of patients sampled and the census counts of messages and staff senders and receivers. Our patient study population included 1031 patients who generated 7346 patient-initiated threads during 2017. Our staff population was 711; of those, 56.6% (403/711) sent and were the intended recipients of at least one message. Our message sample included 18,309 messages, of which slightly more than half (10,163/18,309, 55.55%) were patient-generated.

### Patient Characteristics

We included categorical variables representing patients’ demographic and geography-based characteristics and elements for health status and health care access. Demographic characteristics included age, sex, and race (Black, White, and other). Geography-based characteristics included rural or urban home locations based on rural-urban commuting area [25] codes and average travel distance in miles between clinic and home.

We included patients’ health status markers based on health conditions (ie, diabetes, hypertension, or both conditions) and the number of comorbidities ranging from 1 to 9 from a list of *ICD-10-CM* that frequently occurred within the sampled population. Finally, we incorporated proxy elements for patients’ health care access using payer type (private, public, uninsured, or other) and the number of outpatient visits in 2017. All analyses controlled for the number of threads initiated by patients in 2017 because the greater the number of messages sent by a patient, the greater the opportunity for a variety of taxa.

### Characteristics of Staff

Clinic teams at the medical center typically triage patient messages; thus, the intended recipient was not always the individual who responded to a given message. Our analyses, therefore, differentially identify for each patient-generated message the staff who sent the message response from the receiver as the intended recipient of patient-generated message.

We used two strategies to identify staff receivers as staff to whom the patient intended the message to be delivered. First, for the initial patient-generated message in each thread, we identified the receiver as the staff to whom the message was addressed. Second, we assumed that the receiver for all subsequent patient-generated messages was the sender of the staff-generated message that most recently preceded the patient-generated message. If a staff-generated message did not precede the patient-generated message, we used the same receiver as the most recently preceding patient-generated message.

We included three variables to classify staff. First, staff types were grouped into the 6 most frequently occurring types (ie, administrative staff, licensed practical nurses, nurse practitioners, physicians, registered nurses, and other clinicians). The *other* category included pharmacists, physician assistants, medical assistants, podiatrists, social workers, and medical technicians. Next, we categorized clinical specialty as either primary care or specialty. We included family and internal medicine, geriatrics, pediatrics, obstetrics, and gynecology in our primary care category. Physician assistants, registered nurses, pharmacists, social workers, medical technicians, case managers, counselors, and administrative staff were not assigned a specialty.

Finally, we estimated the message volume for each staff member based on the messages saved to all patients’ charts (not just our sampled population), regardless of whether they were sent in response to a patient-initiated thread or were part of a staff-initiated thread.

### Content Analysis

Consistent with the premises of the Uncertainty in Illness Theory [26] and patient-centered communication [18], our taxonomy includes codes (or taxa) for patients seeking information to alleviate uncertainty around their health status (eg, symptoms and condition) and health care delivery processes. It also includes task-oriented requests that may be used to support self-care or address uncertainty. We included social communication and information-sharing taxa for both patient- and staff-generated messages because these taxa may indicate communication that fosters trust-building between patients and clinicians. For content from staff, the taxonomy also includes action responses based on the taxonomy of requests by patients [27] as leveraged by other researchers. Additional taxa for staff-generated messages classified clinicians’ information-sharing content.

More details on the content analysis process are provided elsewhere [24]; however, in summary, a primary coder read and assigned taxa to all messages, and a second coder did the same for a random 10% sample of messages. Coding units could be no longer than a single message and were frequently shorter, with multiple codes applied to a single message. Each taxon was assigned only once to a given message. We coded the data using NVivo 12 software (QSR International). Discrepancies were reconciled, and the primary coder recoded the messages accordingly.

### Data Analysis

We explored the associations between taxa and characteristics of senders and receivers. For each taxon (ie, individual code), we created a set of dichotomous variables: one set based on the sender and the other on the receiver. For sender-based analyses, we recorded the variable as positive if the patient or staff sent at least one patient-generated or staff-generated message coded with the taxon, respectively. For receiver-based analyses, we assigned a positive value if the patient or staff received at least one staff-generated or patient-generated message coded with the taxon, respectively.

We estimated adjusted odds ratios (ORs) using separate logistic regression models, where each taxon was the dependent variable, and the patient or staff characteristics were the independent variables. Analyses were conducted using version 9.2 of the SAS System for Windows.

## Results

### Population Characteristics

Table 1 shows patient characteristics based on their health conditions. On average, patients sent 9.86 messages (SD 13.70; median 1.0; maximum 117) across 7.12 (SD 9.66) threads. Our population primarily lived in urban areas and comprised approximately two-thirds of women.

Table 2 presents the characteristics of the staff as both receivers and senders. Patients directed more messages to physicians and primary care clinicians. Registered nurses were the most common type of sender staff, followed by physicians. Although 3.69% (376/10,163) of the messages were addressed to administrative staff, those staff accounted for almost a quarter of the messages sent.

**Table 1:** Patient characteristics by health condition (N=1031).

Characteristics			Diabetes only (n=398)		Hypertension only (n=394)		Both conditions (n=239)		Total (N=1031)
Number of messages, mean (SD)			9.93 (13.57)		9.12 (12.91)		10.95 (15.11)		9.86 (13.70)
Number of threads, mean (SD)			7.07 (8.96)		6.54 (9.07)		8.18 (11.51)		7.12 (9.66)
Age (years), mean (SD)			54.65 (13.83)		59.62 (14.34)		60.49 (12.01)		57.91 (13.87)
Distance between home and clinic (miles), mean (SD)			26.88 (38.36)		34.83 (37.62)		31.62 (41.88)		31.02 (39.05)
Number of co-occurring conditions, mean (SD)			3.02 (1.83)		2.95 (1.67)		4.32 (1.88)		3.29 (1.87)
Number of outpatient visits, mean (SD)			14.31 (12.04)		15.54 (12.23)		19.15 (14.29)		15.90 (12.79)
Female, n (%)			277 (69.6)		239 (60.7)		154 (64.4)		670 (65)
Urban home location, n (%)			392 (98.5)		380 (96.4)		233 (97.5)		1005 (97.5)
**Insurance, n (%)**									
	Other	109 (27.4)		87 (22.1)		75 (31.4)		271 (26.3)	
	Private	174 (43.7)		113 (28.7)		44 (18.4)		331 (32.1)	
	Public	108 (27.1)		189 (48)		115 (48.1)		412 (40)	
	Uninsured	7 (1.8)		5 (1.3)		5 (2.1)		17 (1.6)	
**Race, n (%)**									
	Black	182 (45.7)		130 (33.2)		104 (43.5)		416 (40.4)	
	Other	26 (6.5)		12 (3.1)		12 (5)		50 (4.9)	
	White	190 (47.7)		250 (63.8)		123 (51.5)		563 (54.7)	

**Table 2:** Staff characteristics (N=711).

Characteristics			Receivers, n (%)					Senders, n (%)					Total staff (N=711), n (%)
			Staff (n=567)		Messages (n=10,163)		Staff (n=544)			Messages (n=8146)			
**Staff type**													
	Administrative	40 (7.1)		376 (3.7)		79 (14.5)			1927 (23.7)		79 (11.1)		
	Licensed practical nurse	17 (3)		148 (1.5)		32 (5.9)			474 (5.8)		32 (4.5)		
	Nurse practitioner	63 (11.1)		918 (9)		50 (9.2)			503 (6.2)		65 (9.1)		
	Other staff type	27 (4.8)		136 (1.3)		32 (5.9)			158 (1.9)		37 (5.2)		
	Registered nurse	114 (20.1)		1222 (12)		169 (31)			2678 (32.9)		170 (23.9)		
	Physician	294 (51.9)		5736 (56.4)		163 (30)			2380 (29.2)		304 (42.8)		
	Unknown	12 (2.1)		1627 (16)		19 (3.5)			26 (0.3)		24 (3.4)		
**Clinical specialty**													
	N/A^a^	224 (39.5)		1975 (19.4)		324 (59.6)			5176 (63.5)		346 (48.7)		
	Primary	155 (27.3)		4387 (43.2)		116 (21.3)			2002 (24.6)		159 (22.4)		
	Specialty	174 (30.7)		2159 (21.2)		81 (14.9)			937 (11.5)		178 (25)		
	Unknown	14 (2.5)		1642 (16.2)		23 (4.2)			31 (0.4)		28 (3.9)		
**Messages in 2017**													
	≤1000	206 (36.3)		1092 (10.7)		153 (28.1)			479 (5.9)		267 (37.6)		
	1001-2000	134 (23.6)		1320 (13)		134 (24.6)			789 (9.7)		166 (23.3)		
	2001-3400	99 (17.5)		1859 (18.3)		109 (20)			1244 (15.3)		119 (16.7)		
	>3400	109 (19.2)		4240 (41.7)		121 (22.2)			5550 (68.1)		123 (17.3)		
	Unknown	19 (3.4)		1652 (16.3)		27 (5)			84 (1)		36 (5.1)		

^a^N/A: not applicable.

### Patient-Generated Taxa

#### Overview

Table 3 presents a description of each patient-generated taxon, the proportion of patients who sent them, and staff who received these taxa. Most patients sent messages with *information sharing* and *information seeking* content. The lowest percentage of patients sent *social communication* (*appreciation* or *praise*) and preventive care scheduling requests. Furthermore, 3 of 4 staff received at least one *information sharing* and one *information seeking* message. The smallest percentage of staff received appointment cancellation requests.

**Table 3:** Percentage of patient-generated taxa by patient senders and staff recipients.

Patient-generated taxon			Description		Patient senders (N=1031), n (%)		Intended staff recipients (n=567), n (%)
**Information seeking**							
	Logistics	Questions about timing, clinical processes, health care settings, or a patient’s care plan		420 (40.7)		306 (54.0)	
	Medical guidance	Questions that seek medical guidance or information		554 (53.7)		329 (58.0)	
**Information sharing**							
	Sharing clinical update	Sharing information with a clinician that does not require immediate action or response (and may not require action at all)		479 (46.4)		297 (52.4)	
	Self-reporting	Sharing biometrics or other health-related self-measurements; information with a clinician that does not require immediate action or a response		109 (10.6)		66 (11.6)	
	Response to clinician’s message	Response to clinician’s question in a preceding message within the thread		520 (50.4)		258 (45.5)	
**Prescription request**							
	Prescription refill or renewal	Request for prescription refill or renewal		504 (48.9)		246 (43.4)	
	New or change prescription	Request for a new prescription or switch to a different medication or treatment		293 (28.4)		164 (28.9)	
Referral request			Request for a referral to another health care facility or clinician		116 (11.2)		94 (16.6)
Other administrative requests			Process-related and administrative in nature; includes requests for sick notes, contact information, medical records, patient portal access, or information about billing or insurance; technology-related questions; requests for call		312 (30.3)		230 (40.6)
**Scheduling request**							
	Cancellation	Request to cancel an existing appointment with no associated request to change the date or time		213 (20.6)		39 (6.9)	
	Follow-up	Request for an appointment relative to an existing health condition		241 (23.4)		92 (16.2)	
	New condition or symptom	Patient request for an appointment relative to a newly identified health condition or new symptom for an existing condition; new patient appointment		200 (19.4)		76 (13.4)	
	Preventive care	Request for preventive care or routine physical examination		85 (8.2)		17 (3.0)	
	Reschedule	Request for an appointment to be changed to another date or time		393 (38.1)		112 (19.8)	
	Laboratory test or diagnostic procedure	Request for a laboratory test or diagnostic procedure (eg, x-ray or ultrasound) order		145 (14.1)		104 (18.3)	
**Social communication**							
	Appreciation or praise	Content that expresses gratitude or offers acknowledgment or appreciation of service provided, health status, or another act		65 (6.3)		56 (9.8)	
	Complaints	Expressions of frustration or displeasure about service or life issues		96 (9.3)		107 (18.9)	
	Life issues	Communication about aspects of the patients’ life not specifically related to health		125 (12.1)		94 (16.6)	

#### Characteristics of Patients as Senders

**Figure 1:**
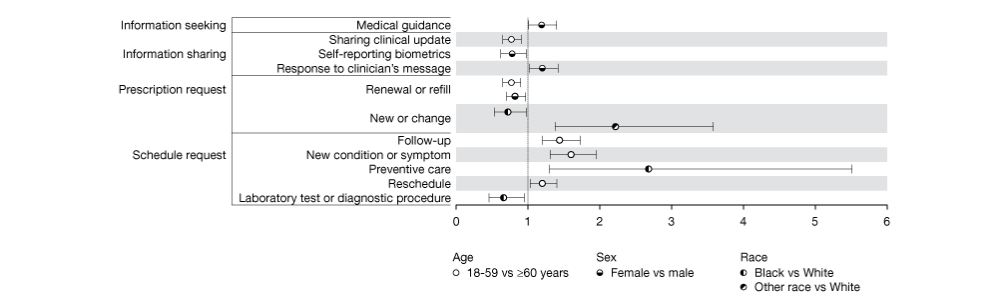
Odds ratios and 95% CIs of associations between patient demographic characteristics and patient-generated message content.

Figure 1 displays the ORs estimated as statistically significant with a *P*<.05 for the associations between patient-generated taxa and patient demographic characteristics. Multimedia Appendix 1 presents the OR estimates for all taxa.

Younger patients had lower odds of sending clinical updates (OR 0.77, 95% CI 0.65-0.91) and prescription refill and renewal requests (OR 0.77, 95% CI 0.65-0.90). They had higher odds of sending scheduling requests, specifically for follow-up appointments (OR 1.44, 95% CI 1.20-1.73), appointments for new conditions or symptoms (OR 1.60, 95% CI 1.31-1.95), and rescheduling (OR 1.20, 95% CI 1.03-1.41). Women had lower odds of self-reporting biometrics (OR 0.78, 95% CI 0.62-0.98) and requesting prescription refills (OR 0.82, 95% CI 0.70-0.97), but higher odds of responding to staffs’ comments or questions (OR 1.20, 95% CI 1.02-1.42) and seeking medical guidance (OR 1.19, 95% CI 1.01-1.40).

Black patients had lower odds of requesting a new or changed medication (OR 0.72, 95% CI 0.53-0.98), scheduling a laboratory or other diagnostic procedure (OR 0.66, 95% CI 0.46-0.95), and requesting an appointment be canceled (OR 0.73, 95% CI 0.53-1.00) compared with White patients. Conversely, Black patients had greater odds (OR 2.68, 95% CI 1.30-5.51) of requesting preventive care appointments than White patients. Patients of other races had greater odds (OR 2.2, 95% CI 1.38-3.58) of requesting a new or changed medication compared with White patients.

**Figure 2:**
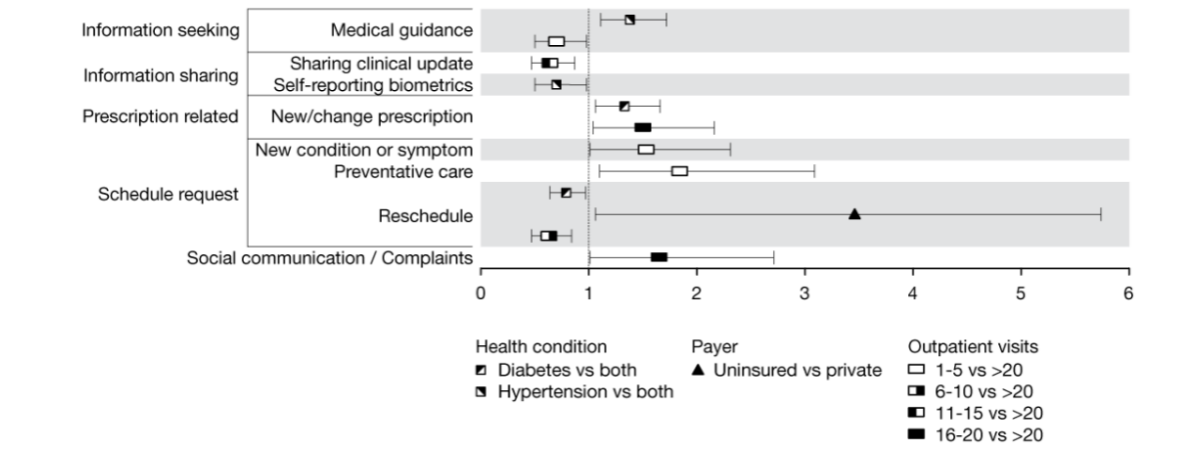
Odds ratios and 95% CIs of associations between patient-generated message content and patient health condition and delivery characteristics.

Figure 2 presents the ORs for patients’ health care access and health status characteristics. Uninsured patients had greater odds (OR 2.46, 95% CI 1.06-5.74) of requesting an appointment to be rescheduled than patients with private payers. Patients with diabetes only had greater odds of requesting a new or changed medication (OR 1.33, 95% CI 1.06-1.66) and reduced odds of requesting an appointment to be rescheduled (OR 0.79, 95% CI 0.64-0.97) compared with patients with both diabetes and hypertension. Patients with hypertension only had greater odds of seeking medical guidance (OR 1.38, 95% CI 1.11-1.72) and reduced odds of self-reporting biometrics (OR 0.70, 95% CI 0.50-0.98) than patients with both conditions.

#### Characteristics of Staff as Receivers

**Figure 3:**
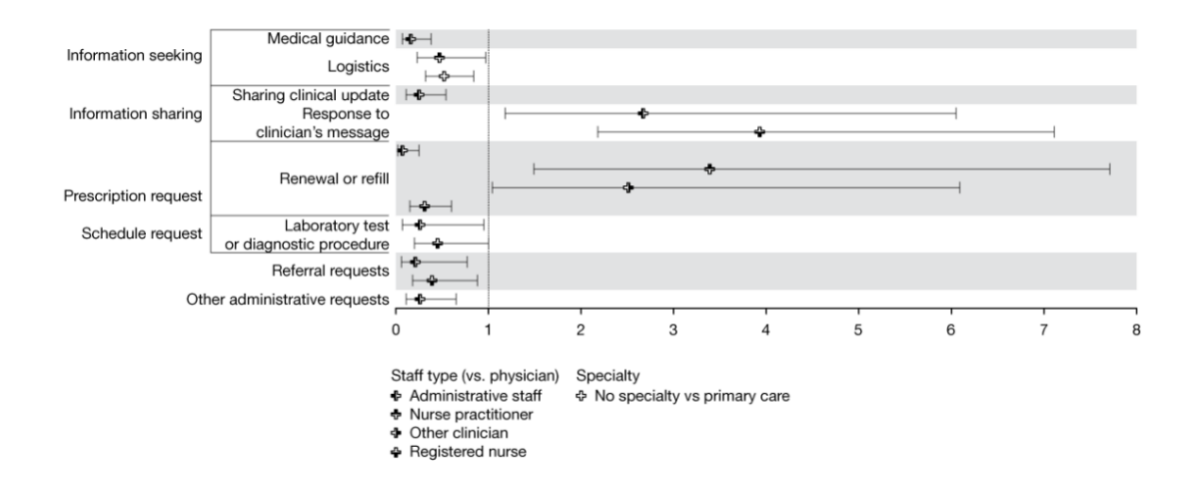
Odds ratios and 95% CIs of associations between clinical staff characteristics and patient-generated content.

An average of 4.92 (SD 7.59) sampled patients sent each staff 15.81 (SD 36.93) messages across 12.92 (SD 27.66) threads. Figure 3 displays the OR estimates of the associations between the characteristics of the staff as receivers of patient-generated taxa. The only differences we observed by specialty were between staff with no applicable specialty and primary care clinicians. Staff not assigned a specialty had lower odds of receiving *logistics* requests (OR 0.52, 95% CI 0.32-0.84) from patients. The administrative staff were less likely to receive medical guidance requests (*P*<.001), clinical updates (*P*<.001), prescription refill requests (*P*<.001), laboratory or other procedure scheduling requests (*P*=.04), and other administrative (*P*=.003) and referral requests (*P*=.02) than physicians. They had greater odds (OR 2.67, 95% CI 1.18-6.05) of receiving responses to their questions. Registered nurses also had greater odds of receiving *response to the clinician’s message* (OR 3.93, 95% CI 2.18-7.11) and lower odds of receiving requests for referrals (*P*=.02) and refilling prescriptions (*P*<.001). Nurse practitioners had greater odds (OR 3.39, 95% CI 1.49-7.71) of receiving prescription-related requests but had lower odds of receiving *logistics* questions (OR 0.47, 95% CI 0.23-0.97) than physicians.

### Staff-Generated Taxa

#### Overview

Table 4 lists the percentages of staff who sent at least one message with the selected taxon. Almost 9 in 10 staff (n=473) shared information with their patients, although only slightly more than half shared medical guidance. More than half of staff sent at least one message that fulfilled a patient’s request.

Three-quarters of the patients (n=798) received at least one message from the staff with information-sharing content. Two-thirds of the patients received message content that fulfilled their request. Few patients received messages that denied their requests or provided encouragement.

**Table 4:** Staff-generated taxa distribution.

Staff-generated taxon			Description		Staff senders (n=544), n (%)		Patient recipients (N=1031), n (%)
**Action response**							
	Acknowledges	Includes recognition that the request for action or information is made, but no indication is provided about whether the request will be fulfilled		148 (27.2)		254 (24.6)	
	Fulfills request	Documentation that the request action was completed		316 (58.1)		686 (66.5)	
	Partially fulfills	Indicates additional steps are necessary to fulfill the request or that only part of the request can or has been completed		161 (29.6)		283 (27.4)	
	Denies request	Indicates that the request will not be fulfilled		57 (10.5)		95 (9.2)	
Information seeking			Clinicians’ requests for information or clarity around patients’ condition or symptoms, or symptom severity or duration		248 (45.6)		552 (53.5)
Deferred information sharing			Clinical responses that refer the patient to another clinician for a response; postpone an answer pending additional clinical information		248 (45.6)		503 (48.8)
**Information sharing**							
	Medical guidance	Provides treatment decisions, gives care instructions, dietary guidance, instructs the patient on the best next steps in their care plan, interprets diagnostic procedure or laboratory results, or provides information on symptoms or the patient’s health condition		299 (55)		503 (48.8)	
	Orientation to procedures, treatments, or preventive behaviors	Explains what a patient might expect during treatment or diagnostic procedure or in a new health care setting or situation		371 (68.2)		718 (69.6)	
Recommendation to schedule an appointment			Suggestion that patient schedule an appointment		113 (20.8)		170 (16.5)
Social communication or encouragement			Provides positive reinforcement of patient’s actions or behaviors		38 (7)		58 (5.6)

#### Characteristics of Staff as Senders

**Figure 4:**
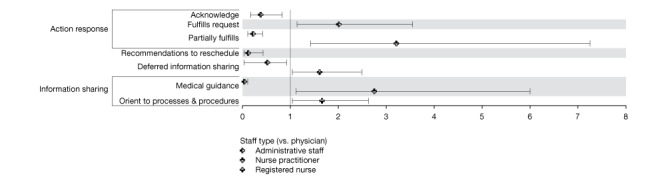
Odds ratios and 95% CIs of associations between clinical staff characteristics and staff-generated content.

Staff responded, on average, with 15.47 (SD 41.12) messages to 11.60 (SD 30.69) threads initiated by 6.69 (SD 17.83) patients. Figure 4 presents the estimates of the associations between taxa and staff characteristics. We observed no associations between taxa and clinical specialty after controlling for staff type and message volume. Administrative staff had reduced odds of sending many taxa compared with physicians, except for *fulfills request* (OR 2.01, 95% CI 1.14-3.55). Nurse practitioners had greater odds (OR 2.74, 95% CI 1.12-6.68) of sharing medical guidance with patients and greater odds (OR 3.21, 95% CI 1.42-7.25) of partially fulfilling patients’ requests compared with physicians. Registered nurses also had greater odds of deferred information sharing (OR 1.61, 95% CI 1.04-2.49) and *orientation to procedures, treatments, and preventive behaviors* (OR 1.66, 95% CI 1.04-2.63) compared with physicians.

#### Characteristics of Patients as Receivers

**Figure 5:**
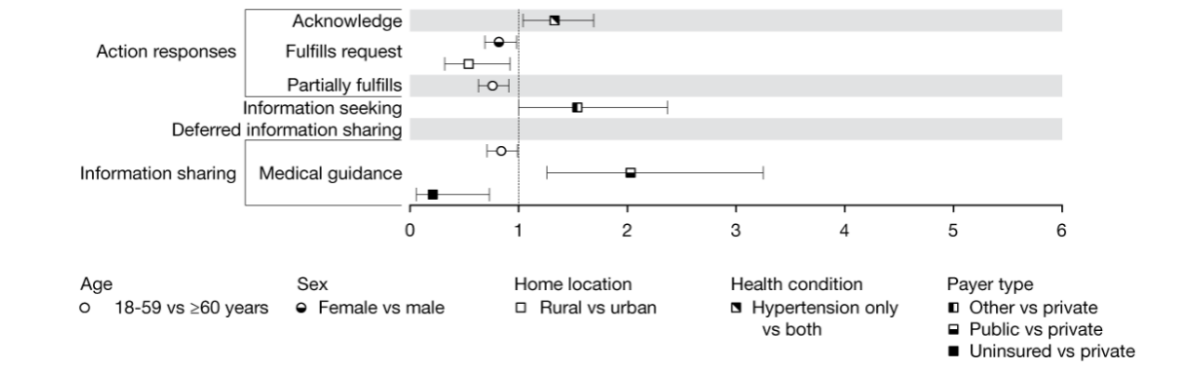
Odds ratios and 95% CIs of associations between patient characteristics and staff-generated content.

Figure 5 displays the OR estimates for associations between clinician-generated taxa and the characteristics of patients who received these taxa. Younger patients had reduced odds of receiving partial request fulfillment (OR 0.76, 95% CI 0.63-0.91) and medical guidance (OR 0.84, 95% CI 0.71-0.99). Women and individuals with a rural home address had reduced odds (OR 0.82, 95% CI 0.69-0.98 and OR 0.54, 95% CI 0.32-0.92, respectively) of receiving confirmation that their requests were fulfilled. Patients with public payers had more than twofold increased odds (OR 2.03, 95% CI 1.26-3.25), whereas uninsured patients had reduced odds (OR 0.21, 95% CI 0.06-0.73) of receiving medical guidance compared with patients with private payers.

## Discussion

### Principal Findings

#### Overview

As expected, secure message content varied based on the characteristics of both the sender and receiver. The patients’ message content varied based on age, sex, home location, insurance type, and health condition. Staff-generated content varied primarily based on staff type. Message content staff sent to patients varied based on the patients’ age, sex, health condition, and payer status. Finally, patients sent different content based on staff type. Given that other research demonstrated that secure message content was associated with selected health outcomes [28], our findings may indicate that inequitable use of secure messaging could further compound existing disparities in health care delivery and outcomes.

#### Patient-as-Sender

Patients who trust their clinicians may be more open to sharing information with their clinicians [17]. Previous research reported a positive association between patients’ age and trust in their clinicians [29] and found that non-Hispanic White patients and men were more likely to disclose information to clinicians [30]. Consistent with these findings, we observed that younger patients were less likely to share clinical updates with their clinical team, and women were less likely than men to self-report biometrics through secure messaging. As sharing relevant clinical information with the care team can be important to the continuity of care and ongoing patient engagement, it will be important to better understand why these populations might not be taking advantage of secure messaging in this way.

Our findings indicated no difference by race for information-seeking and information-sharing content, contrary to other studies that found that Black and other race patients reported higher levels of trust in other information sources (eg, charitable organizations, newspapers, and radio) [31]. As there is an existing divide by race in the use of secure messaging [14-16], there may be less observable differences by race in the content among the patients who opt to use secure messaging. That is, patients who opt to use secure messaging are those who trust their clinic staff to provide the information they need more so than those other sources. Further research should explore the association of trust among users of secure messaging.

Our study found that Black patients were less likely to request changes to their prescriptions or request laboratory or other diagnostic procedures, whereas patients of other races were more likely to request prescription changes than White patients. These 2 request types, unlike some other task-oriented request taxa, involve a more active involvement from the patient to be aware of a medical need and outreach to the clinician to request clinical action for a change in care. Two-thirds of studies in a literature review of the effects of race on patient-physician communication reported that Black patients had fewer acts of participation during their physician visits [32]. If requests for a new or changed medication, laboratory, or other diagnostic procedures are considered more participatory in nature, then our research extends these findings to electronic communication modalities.

#### Staff-as-Receiver

Differences in the types of messages sent by staff were likely reflective of the fact that many practices triage messages through a team of nurses, physician assistants, pharmacists, and physicians, with physicians generally responding only to the more complicated messages [33-35]. We observed that patients were more likely to send prescription requests to nurse practitioners than physicians and were more likely to send referral and laboratory and diagnostic procedure requests to physicians, which is consistent with a triage response system.

As expected, patients intended most of their information-seeking messages to be received by physicians, nurse practitioners, and registered nurses. Although there were no differences by staff type for the staff-generated *information seeking* taxon, patients were almost four times more likely to send *responses to clinician’s messages* to registered nurses and three times more likely to send them to administrative staff. This could indicate one of two factors: (1) those staff types asked more questions about patients or (2) those staff types were better at soliciting responses from patients. As noted previously, patient information sharing is a marker of trust with the clinical provider, so higher occurrences of the patient and clinical team engaging in electronic bidirectional dialog represented by this taxon might lead to strong trust or be a marker of existing trust. Alternatively, registered nurses and administrative staff sent high volumes of secure messages, so they may be more comfortable with the communication modality and better able to ask questions in a manner in which the patient is comfortable. Future studies should incorporate experience with secure messaging to control for this potential confounder.

#### Staff-as-Sender

Consistent with the triaging process, we found that administrative staff were less likely than physicians to share information and make recommendations to schedule appointments. In a triage system where physicians generally respond to the most complex messages, it makes sense that registered nurses and nurse practitioners were more likely than physicians to send most types of messages, as our data showed.

Previous research showed that almost 2 out of 10 office visits with a primary care physician were suitable for another modality [36]. Our research demonstrates that much of information sharing and action responses to messages is handled by registered nurses and nurse practitioners, although physicians still send the second highest number of messages. As messages could be coded with more than one taxon, it is possible that nurse respondents sent messages that addressed more than one content area, compared with physicians whose responses may have been more targeted.

#### Patient-as-Receiver

Younger patients were less likely to receive acknowledgment and indications for partial fulfillment. We observed differences according to age for patients’ task-oriented requests (eg, scheduling, prescription-related, and administrative requests), although directionality varied (eg, younger patients were more likely to send scheduling requests but less likely to make prescription requests). It may be that the difference in action responses from the staff was associated with the preceding request type. It is unclear whether these data represent differential fulfillment rates by request type or a difference in the way staff communicate based on patient age.

Similarly, although we identified only one difference by patient sex associated with sending task-oriented requests, women were less likely to receive fulfillment responses. Further research is needed to determine if differences in fulfillment rates are based on patients’ sex or the nature of the requests made by the patient. Research that explores the differences in responses among subsets of patients who sent messages with selected taxa could determine whether these responses vary among patients requesting that type of information. For example, do staff respond to prescription requests differently based on patient characteristics, whereas scheduling requests receive standard responses regardless of patient demographics? Our research did not explore the paired call-response nature of the secure message thread. Future research should explore the best approach to analyzing paired taxa in threads to understand the associations between a patient request and the staff response to that request.

The staffs’ message responses did not vary by patient race. The literature on differences in patient-clinician communication by race is mixed, but a recent literature review found that most vignette studies detected no association between clinicians’ implicit bias and treatment recommendations [37]. A small observational study found no differences in verbal communication by race but higher nonverbal communication scores for White patients [38]. Conversely, another review noted that 5 of 6 observational and patient-reported measure-based studies found that physicians provided Black patients with less information than White patients [32]. The fact that our study found no differences in messages sent to patients by race may be because the taxonomy is based solely on the text in the message and does not leverage any nonverbal cues. Research has found evidence that nonverbal cues in text-based messages (eg, differential use of upper- and lower-case letters, spelling and grammar errors, and emoticons) can affect receivers’ assessment of the senders’ competence and change receivers’ interpretation of the emotional intent of the message [39,40]. Thematic coding of secure messages found tone mismatches in about 16% (11/70) of the messages reviewed [41]; such mismatches could reduce patient engagement and limit patients’ understanding and acceptance of any guidance provided. Comparison of message content through the more objective lens of this taxonomy coupled with a more subjective evaluation of message tone and nonverbal cues may help determine whether there are more subjective differences in message content by race or other characteristics.

Sharing medical guidance from clinicians varied by patient payer type; compared with patients with private payers, staff were more likely to send messages with medical guidance to patients with public payer types and less likely to send that content to uninsured patients. An analysis of the Medical Expenditure Panel Survey found that patients without insurance—compared with patients with public insurance—were less likely to report that their provider always listened and explained things in a way they understood [42]. Our study’s findings may be an indicator from the electronic communication medium perspective of why patients without insurance might report those perceptions.

### Limitations

This study was based on messages saved to patient charts because they were available for extraction at the time of this study. This means that messages sent by patients and any responses not saved to patient charts were not part of the analysis. We have no way to determine if there were trends by staff characteristics in saving messages to patient charts; therefore, we have no way to estimate whether this would further affect the associations we observed between taxa and patient and staff characteristics.

Our analysis did not include communication between patients and clinicians that occurred outside of the sampled patient-initiated threads. It is possible that a patient, for example, initiated a conversation with a clinician via phone that was concluded by a clinician-initiated thread or that a clinician responded to a patient’s secure message with a phone call. We did not capture these examples and others that would fall outside of patient-initiated secure message communications. Little research has been done to explore the frequency of such cross-communications, but a small study found that approximately half of the patients’ unanswered threads were resolved through other mechanisms [41]. Patient care, however, should be provided in the form needed by the patient and be responsive to patient choices and preferences [43]. If patients opt to communicate with their staff via secure messaging, it is likely that patients desire a response through that communication modality. A response through another modality may not demonstrate the best patient-centered practices. Future studies should explore whether communication occurred through other modalities and what those responses were, to better understand whether there are certain contexts when a response through an alternate modality might be appropriate, and which populations benefit from communication modality shifts.

We were also missing 5.2% (37/711) of data on staff, but that translated to 16.2% (1647/10,163) of messages not included in analyses that used staff characteristics. It is again difficult to understand the impact of this loss of data on the overall trends, but our unadjusted comparisons of the staff with unknown characteristics indicated likely within-group differences.

Finally, our cut-point values for our continuous variables were based on sample distributions. Future analyses should conduct sensitivity analyses to determine the best distribution of those cut-point values.

### Conclusions

Our research presents the first analysis that associates the differences between message content and patient and staff characteristics. It demonstrates clear differences in the secure messaging content patients and staff used based not only on their respective characteristics but also those of the individuals with whom they communicated. It is important to recognize that similar to in-person communication, differences exist in communication patterns based on patient and staff characteristics. The differences we found in content use based on patient characteristics could lead to the exacerbation of health disparities when content is associated with those health outcomes. Creative technological solutions may be necessary to mitigate these differences; for example, natural language processing could be used to standardize some queries and responses and provide patients and staff with suggested text to improve communication. In the absence of a technological solution, staff and administrators should evaluate how secure messaging is used to ensure that disparities in care are not perpetuated via this communication modality.

## Appendix

Multimedia Appendix 1 Sampled data frequencies and odds ratios (95% CIs) estimating the association between characteristics and secure message content.

## Abbreviations

*ICD-10-CM*: *International Classification of Diseases, Tenth Revision, Clinical Modification*
OR: odds ratio
