# Supplementary material for: Patient and Clinician Characteristics Associated With Secure Message Content: Retrospective Cohort Study
Source: J Med Internet Res. 2021 Aug 19;23(8):e26650. doi: 10.2196/26650 (PMC8414300; doi:10.2196/26650)
Supplement: Multimedia Appendix 1 [file jmir_v23i8e26650_app1.pdf]

Patient and Clinician Characteristics Associated with Secure Message Content: Retrospective Cohort

Multimedia Appendix 1.

Table S1. Number and Percent of Sampled Patients by Health Condition

	Diabetes only	Hypertension only	Both diabetes and hypertension	Total
Patients who met health condition inclusion criteria and who were registered with the patient portal	683	3,546	2,503	6,732
Patients who initiated a message thread in 2017, n (%)	398 (58.3)	1,607 (45.3)	1,132 (45.2)	3,137 (46.6)
Sampled patients, n (% of patients who initiated a message thread)	398 (100)	394 (24.5)	239 (21.1)	1,031 (32.9)
Total messages sent by patients	3,953	3,592	2,618	10,163
Total unique clinic staff who responded to sampled patients' messages*	344	340	293	544
Total unique clinic staff who received sampled patients' messages*	366	365	285	567
Total message responses sent by clinic staff	3,138	2,917	2,091	8,146

*Unique senders or receivers by category; some clinicians received or responded to patients across categories.

Approved for Public Release; Distribution Unlimited. Public Release Case Number 20-3387 The author's affiliation with The MITRE Corporation is provided for identification purposes only and is not intended to convey or imply MITRE's concurrence with, or support for, the positions, opinions, or viewpoints expressed by the author. ©2020 The MITRE Corporation. ALL RIGHTS RESERVED

Table S2. Odds Ratios (95% Confidence Intervals) Estimating the Association between Patient Characteristics and Patient-Generated Message Content

Patient Characteristics	Information seeking		Information sharing			Task-oriented requests								Other Administrative	Social communication		
						Prescription-related		Scheduling									
	Logistics	Medical guidance	Sharing clinical update	Self-reporting biometrics	Response to clinician's msg	Prescription refills/renewals	New/change prescription	Cancellation	Follow-up	New condition or symptom	Preventive care	Reschedule	Laboratory test or diagnostic procedure		Appreciation/ praise	Complaints	Life issues
18-59 years vs 60+ years	1.05 (0.89-1.25)	0.85 (0.72-1.00)	0.77 (0.65-0.91)	0.97 (0.77-1.23)	1.01 (0.85-1.20)	0.77 (0.65-0.90)	1.08 (0.91-1.29)	0.89 (0.74-1.07)	1.44 (1.20-1.73)	1.60 (1.31-1.95)	1.16 (0.89-1.51)	1.20 (1.03-1.41)	1.09 (0.87-1.35)	0.97 (0.82-1.15)	1.00 (0.73-1.36)	1.16 (0.89-1.50)	1.03 (0.81-1.30)
Female vs Male	1.11 (0.94-1.31)	1.19 (1.01-1.40)	1.04 (0.89-1.23)	0.78 (0.62-0.98)	1.20 (1.02-1.42)	0.82 (0.70-0.97)	1.01 (0.84-1.20)	1.12 (0.94-1.35)	0.93 (0.78-1.12)	1.00 (0.83-1.22)	1.11 (0.84-1.46)	0.98 (0.84-1.15)	0.97 (0.78-1.20)	1.02 (0.86-1.21)	0.91 (0.68-1.23)	0.97 (0.75-1.26)	0.87 (0.70-1.10)
**Race (vs White)**																	
Black	0.99 (0.73-1.34)	1.01 (0.75-1.36)	0.95 (0.70-1.28)	1.22 (0.80-1.86)	1.15 (0.85-1.56)	1.14 (0.86-1.51)	0.72 (0.53-0.98)	0.73 (0.53-1.00)	1.14 (0.82-1.59)	1.09 (0.78-1.52)	2.68 (1.30-5.51)	1.12 (0.84-1.48)	0.66 (0.46-0.95)	0.82 (0.61-1.11)	1.12 (0.61-2.05)	0.88 (0.54-1.43)	1.10 (0.67-1.80)
Other race	0.81 (0.50-1.31)	0.88 (0.55-1.42)	1.08 (0.67-1.72)	0.85 (0.43-1.65)	0.70 (0.43-1.15)	1.02 (0.65-1.60)	2.22 (1.38-3.58)	1.40 (0.87-2.25)	0.78 (0.45-1.34)	0.97 (0.56-1.66)	0.33 (0.09-1.26)	1.11 (0.70-1.74)	1.60 (0.95-2.69)	1.35 (0.84-2.16)	0.71 (0.26-1.94)	0.94 (0.44-2.03)	0.57 (0.25-1.31)
**Health condition (vs Both)**																	
Diabetes	0.93 (0.75-1.16)	0.99 (0.80-1.24)	1.19 (0.96-1.49)	1.30 (0.97-1.75)	0.94 (0.75-1.18)	1.11 (0.90-1.37)	1.33 (1.06-1.66)	0.95 (0.75-1.21)	1.09 (0.87-1.38)	1.00 (0.78-1.28)	0.8 (0.57-1.13)	0.79 (0.64-0.97)	1.18 (0.89-1.55)	1.17 (0.94-1.46)	1.14 (0.78-1.68)	1.30 (0.93-1.83)	1.00 (0.74-1.36)
Hypertention	1.00 (0.80-1.24)	1.38 (1.11-1.72)	1.04 (0.84-1.29)	0.70 (0.50-0.98)	1.02 (0.82-1.27)	0.86 (0.70-1.06)	0.82 (0.65-1.03)	0.86 (0.67-1.09)	0.98 (0.77-1.25)	0.97 (0.75-1.25)	1.02 (0.72-1.44)	1.13 (0.92-1.39)	0.86 (0.64-1.16)	0.92 (0.73-1.15)	0.88 (0.58-1.31)	1.37 (0.97-1.95)	1.27 (0.94-1.72)
Non-urban vs Urban	1.12 (0.67-1.88)	1.02 (0.61-1.72)	1.38 (0.81-2.34)	1.42 (0.71-2.83)	1.04 (0.62-1.77)	0.80 (0.48-1.34)	0.89 (0.50-1.61)	0.65 (0.34-1.25)	0.57 (0.26-1.24)	1.18 (0.66-2.09)	0 (0-1.544673365	0.78 (0.47-1.29)	1.05 (0.53-2.10)	1.13 (0.67-1.89)	1.32 (0.58-3.02)	1.38 (0.68-2.83)	0.70 (0.32-1.57)
**Payer Type (vs Private payer)**																	
Other payer	0.98 (0.65-1.48)	0.99 (0.67-1.47)	1.22 (0.81-1.83)	0.97 (0.52-1.83)	1.26 (0.82-1.93)	0.96 (0.66-1.39)	1.08 (0.67-1.73)	0.89 (0.60-1.33)	1.26 (0.79-2.00)	1.04 (0.69-1.57)	1.12 (0.58-2.18)	0.94 (0.66-1.35)	0.98 (0.52-1.82)	1.40 (0.92-2.12)	0.76 (0.37-1.56)	0.69 (0.39-1.21)	1.29 (0.67-2.48)
Public payer	1.26 (0.85-1.86)	1.25 (0.86-1.83)	1.33 (0.90-1.98)	0.95 (0.52-1.76)	1.17 (0.77-1.77)	1.05 (0.73-1.50)	1.40 (0.88-2.22)	0.71 (0.48-1.05)	0.92 (0.58-1.46)	0.90 (0.60-1.36)	1 (0.52-1.93)	0.71 (0.50-1.01)	1.12 (0.61-2.06)	1.08 (0.72-1.62)	1.50 (0.78-2.88)	0.96 (0.56-1.65)	1.26 (0.66-2.39)
Uninsured	0.72 (0.27-1.92)	0.58 (0.23-1.48)	0.54 (0.20-1.44)	0.80 (0.17-3.86)	0.46 (0.16-1.33)	0.73 (0.31-1.72)	0.52 (0.16-1.74)	1.36 (0.55-3.38)	0.74 (0.23-2.39)	1.67 (0.64-4.34)	0.65 (0.13-3.3)	2.46 (1.06-5.74)	0.63 (0.13-3.14)	0.71 (0.26-1.96)	1.56 (0.31-7.84)	2.72 (0.75-9.91)	0.56 (0.10-3.05)
**Number of threads intiated (vs 8+)**																	
1	0.20 (0.13-0.30)	0.22 (0.16-0.31)	0.25 (0.18-0.36)	0.45 (0.23-0.86)	0.14 (0.09-0.21)	0.26 (0.19-0.36)	0.21 (0.12-0.35)	0.36 (0.22-0.59)	0.33 (0.20-0.53)	0.29 (0.16-0.51)	0.25 (0.11-0.59)	0.36 (0.25-0.51)	0.17 (0.07-0.44)	0.24 (0.15-0.39)	0.29 (0.09-0.94)	0.12 (0.03-0.62)	0.50 (0.25-0.98)
2	0.44 (0.30-0.64)	0.45 (0.32-0.62)	0.46 (0.33-0.65)	0.53 (0.26-1.06)	0.60 (0.44-0.83)	0.61 (0.44-0.84)	0.56 (0.36-0.88)	0.83 (0.54-1.28)	0.76 (0.49-1.15)	0.54 (0.33-0.91)	0.65 (0.33-1.27)	0.50 (0.35-0.72)	0.49 (0.24-1.01)	0.59 (0.39-0.89)	0.61 (0.22-1.64)	0.69 (0.27-1.71)	0.27 (0.11-0.71)
3-4	0.90 (0.67-1.21)	0.97 (0.74-1.28)	0.70 (0.52-0.93)	0.98 (0.59-1.64)	0.83 (0.62-1.10)	0.87 (0.66-1.14)	1.02 (0.72-1.44)	0.87 (0.60-1.27)	0.84 (0.58-1.22)	0.97 (0.65-1.43)	1.01 (0.58-1.76)	1.26 (0.95-1.67)	1.17 (0.71-1.91)	0.96 (0.70-1.34)	1.10 (0.55-2.22)	0.83 (0.38-1.78)	0.91 (0.53-1.58)
5-7	1.97 (1.48-2.62)	1.45 (1.10-1.93)	1.99 (1.50-2.64)	1.43 (0.90-2.27)	1.72 (1.29-2.29)	1.48 (1.13-1.96)	1.74 (1.26-2.39)	1.36 (0.97-1.92)	1.28 (0.92-1.80)	1.55 (1.08-2.24)	1.82 (1.13-2.92)	1.27 (0.95-1.68)	2.13 (1.35-3.34)	1.45 (1.07-1.98)	1.25 (0.62-2.53)	2.14 (1.12-4.07)	1.25 (0.75-2.10)
**Distance to clinic in miles (vs 40+)**																	
0-8	0.72 (0.54-0.95)	0.87 (0.66-1.15)	0.69 (0.52-0.91)	0.85 (0.57-1.26)	0.91 (0.69-1.20)	1.25 (0.96-1.63)	1.05 (0.79-1.39)	0.82 (0.60-1.11)	1.34 (1.01-1.77)	0.92 (0.67-1.26)	0.84 (0.56-1.28)	1.06 (0.82-1.37)	0.88 (0.61-1.27)	0.86 (0.65-1.14)	0.90 (0.55-1.48)	0.90 (0.59-1.39)	0.90 (0.62-1.31)
9-16	1.30 (1.00-1.68)	1.08 (0.83-1.39)	1.15 (0.89-1.49)	1.19 (0.82-1.73)	1.47 (1.13-1.91)	0.91 (0.71-1.17)	1.12 (0.86-1.48)	0.81 (0.60-1.09)	1.09 (0.82-1.45)	1.19 (0.89-1.61)	0.94 (0.63-1.42)	0.97 (0.75-1.24)	1.00 (0.70-1.41)	1.14 (0.87-1.48)	1.06 (0.64-1.74)	1.13 (0.75-1.72)	0.90 (0.61-1.32)
17-39	1.01 (0.78-1.32)	1.10 (0.84-1.44)	1.26 (0.97-1.64)	1.46 (1.02-2.08)	0.99 (0.75-1.29)	0.82 (0.63-1.06)	0.98 (0.74-1.30)	1.07 (0.81-1.43)	0.90 (0.67-1.20)	1.22 (0.90-1.65)	1.31 (0.87-1.97)	0.95 (0.73-1.22)	1.30 (0.94-1.80)	0.93 (0.70-1.22)	1.21 (0.76-1.93)	1.26 (0.85-1.87)	1.23 (0.86-1.74)
**Number of co-occurring conditions (vs 6-9)**																	
1	1.32 (0.93-1.88)	1.02 (0.73-1.45)	1.23 (0.87-1.74)	1.00 (0.6-1.67)	1.09 (0.76-1.55)	0.81 (0.58-1.13)	0.94 (0.64-1.38)	0.89 (0.59-1.33)	0.73 (0.48-1.09)	0.78 (0.50-1.20)	1.28 (0.76-2.17)	1.01 (0.72-1.41)	1.36 (0.86-2.15)	0.99 (0.68-1.44)	1.03 (0.49-2.17)	0.86 (0.47-1.58)	1.23 (0.76-2.00)
2	0.89 (0.67-1.19)	1.06 (0.80-1.42)	0.88 (0.66-1.17)	0.82 (0.54-1.26)	1.10 (0.82-1.48)	0.88 (0.67-1.16)	1.00 (0.74-1.36)	1.00 (0.73-1.37)	0.99 (0.72-1.36)	0.87 (0.61-1.22)	1.01 (0.64-1.58)	1.02 (0.78-1.34)	1.41 (0.99-2.01)	1.08 (0.81-1.46)	1.04 (0.59-1.83)	1.08 (0.68-1.71)	0.96 (0.64-1.42)
3-5	1.05 (0.83-1.33)	0.93 (0.74-1.19)	0.91 (0.72-1.16)	1.02 (0.73-1.42)	0.79 (0.61-1.00)	1.16 (0.92-1.46)	0.84 (0.66-1.08)	1.02 (0.79-1.33)	1.21 (0.94-1.56)	1.17 (0.90-1.54)	0.85 (0.58-1.24)	0.89 (0.71-1.12)	0.68 (0.50-0.93)	1.01 (0.80-1.29)	1.12 (0.72-1.72)	0.92 (0.63-1.33)	0.69 (0.50-0.96)
**Number of outpatient visits (vs 21+)**																	
1-5	0.93 (0.66-1.32)	0.70 (0.50-0.98)	0.94 (0.67-1.33)	0.89 (0.51-1.54)	0.87 (0.62-1.23)	1.21 (0.87-1.68)	0.77 (0.52-1.14)	0.76 (0.50-1.16)	0.98 (0.66-1.47)	1.53 (1.01-2.31)	1.84 (1.1-3.09)	1.21 (0.87-1.69)	0.84 (0.52-1.37)	0.92 (0.64-1.34)	0.63 (0.26-1.52)	0.77 (0.39-1.50)	1.06 (0.62-1.81)
6-10	0.99 (0.74-1.33)	0.90 (0.68-1.19)	1.11 (0.84-1.49)	1.43 (0.94-2.16)	1.02 (0.76-1.36)	0.99 (0.75-1.31)	0.85 (0.62-1.17)	0.95 (0.68-1.32)	1.08 (0.78-1.49)	1.06 (0.74-1.51)	1.22 (0.78-1.92)	0.63 (0.47-0.84)	0.77 (0.51-1.17)	0.94 (0.70-1.28)	1.24 (0.71-2.16)	0.76 (0.45-1.31)	1.04 (0.67-1.62)
11-15	0.88 (0.65-1.20)	1.17 (0.86-1.59)	0.64 (0.47-0.87)	0.73 (0.45-1.20)	1.34 (0.98-1.84)	0.94 (0.70-1.27)	0.93 (0.68-1.29)	1.05 (0.75-1.48)	1.00 (0.71-1.40)	1.01 (0.70-1.44)	0.86 (0.52-1.43)	1.14 (0.85-1.52)	0.81 (0.53-1.24)	0.80 (0.58-1.11)	1.04 (0.58-1.87)	0.85 (0.50-1.42)	1.19 (0.78-1.83)
16-20	1.16 (0.81-1.67)	1.21 (0.83-1.76)	1.13 (0.78-1.63)	0.88 (0.52-1.49)	0.89 (0.61-1.29)	0.94 (0.66-1.34)	1.50 (1.04-2.16)	1.39 (0.96-2.03)	0.95 (0.65-1.40)	0.72 (0.47-1.10)	0.97 (0.56-1.68)	1.22 (0.87-1.71)	1.32 (0.85-2.07)	1.32 (0.92-1.89)	0.92 (0.46-1.84)	1.65 (1.01-2.71)	0.78 (0.47-1.31)

Notes: Each column represents a separate logistic regression model for which the dependent variable is the column header and the rows represent the independent variables. Model for *Referral requests* did not converge.

Approved for Public Release; Distribution Unlimited. Public Release Case Number 20-3387 The author's affiliation with The MITRE Corporation is provided for identification purposes only and is not intended to convey or imply MITRE's concurrence with, or support for, the positions, opinions, or viewpoints expressed by the author.
©2020 The MITRE Corporation. ALL RIGHTS RESERVED

Table S3. Odds Ratios (95% Confidence Intervals) Estimating the Association between Clinic Staff Characteristics and Patient-Generated Message Content

Clinic staff characteristics	Information seeking		Information sharing		Task-oriented requests				Other Administrative	Referral request	Social communication
					Prescription-related						
	Logistics	Medical guidance	Sharing clinical update	Response to clinician's msg	Prescription refills/renewals	Schedule request	Reschedule	Laboratory test or diagnostic procedure			Complaints
**Staff type (vs Physician)**											
Administrative staff	1.08 (0.55-2.11)	0.16 (0.07-0.39)	0.25 (0.11-0.54)	2.67 (1.18-6.05)	0.07 (0.02-0.25)	0.96 (0.49-1.88)	1.96 (0.91-4.25)	0.26 (0.07-0.95)	0.26 (0.11-0.65)	0.21 (0.06-0.77)	0.48 (0.15-1.47)
Licensed practical nurse	1.71 (0.68-4.29)	0.8 (0.32-2.02)	1.1 (0.45-2.7)	1.26 (0.47-3.38)	0.97 (0.34-2.76)	0.66 (0.25-1.72)	0.52 (0.14-1.96)	1.25 (0.39-4.06)	0.94 (0.36-2.47)	1.71 (0.57-5.13)	1.5 (0.46-4.83)
Nurse Practitioner	0.47 (0.23-0.97)	1.71 (0.79-3.7)	1.79 (0.87-3.68)	0.5 (0.23-1.12)	3.39 (1.49-7.71)	1.77 (0.86-3.63)	2 (0.82-4.9)	1.94 (0.79-4.81)	1.63 (0.78-3.39)	1.49 (0.58-3.87)	1.16 (0.46-2.92)
Other clinician	1.44 (0.69-3.04)	1.27 (0.57-2.81)	1.05 (0.49-2.26)	0.47 (0.2-1.1)	2.56 (1.06-6.21)	0.97 (0.43-2.16)	0.55 (0.16-1.97)	1.43 (0.46-4.4)	1.05 (0.45-2.47)	1.49 (0.48-4.66)	0.74 (0.21-2.65)
Registered Nurse	0.96 (0.59-1.56)	0.69 (0.41-1.16)	0.79 (0.49-1.3)	3.93 (2.18-7.11)	0.31 (0.16-0.61)	0.53 (0.31-0.89)	0.61 (0.31-1.2)	0.45 (0.2-1)	1.18 (0.7-1.98)	0.39 (0.18-0.88)	0.85 (0.42-1.73)
**Clinical speciality (vs Primary care)**											
Not applicable	0.52 (0.32-0.84)	0.63 (0.39-1.01)	0.75 (0.48-1.18)	0.76 (0.43-1.35)	0.64 (0.4-1.03)	0.77 (0.48-1.22)	0.87 (0.47-1.61)	0.76 (0.41-1.41)	0.65 (0.4-1.04)	1.02 (0.55-1.89)	0.66 (0.35-1.25)
Specialist	1.1 (0.78-1.54)	1.35 (0.94-1.96)	1.03 (0.74-1.43)	1.03 (0.69-1.54)	0.84 (0.6-1.19)	1.16 (0.84-1.62)	1.47 (0.97-2.24)	0.97 (0.64-1.49)	1.18 (0.84-1.65)	0.75 (0.48-1.16)	1.11 (0.73-1.69)
**Number of messages sent in 2017 (vs >3400)**											
<=1000	0.44 (0.32-0.59)	0.29 (0.2-0.42)	0.51 (0.37-0.69)	0.32 (0.23-0.46)	0.29 (0.2-0.43)	0.43 (0.31-0.58)	0.38 (0.25-0.57)	0.35 (0.23-0.52)	0.46 (0.33-0.62)	0.33 (0.21-0.51)	0.46 (0.31-0.68)
1001-2000	0.86 (0.63-1.18)	0.77 (0.53-1.11)	0.64 (0.46-0.88)	0.55 (0.39-0.79)	0.62 (0.42-0.92)	0.88 (0.64-1.21)	0.85 (0.58-1.25)	0.71 (0.47-1.06)	0.75 (0.54-1.04)	0.67 (0.43-1.04)	1.17 (0.81-1.7)
2001-3400	1.16 (0.81-1.65)	1.74 (1.13-2.69)	1.11 (0.77-1.6)	1.6 (1.09-2.34)	1.13 (0.73-1.75)	1.24 (0.87-1.77)	1.36 (0.91-2.03)	1.63 (1.08-2.45)	1.3 (0.91-1.85)	1.38 (0.9-2.11)	0.91 (0.59-1.4)

Note: Each column represents a separate logistic regression model for which the dependent variable is the column header and the rows represent the independent variables. Models for self-reporting biometrics, new or changed prescription requests, appointment cancellation requests, follow-up appointment requests, requests for appointments for new conditions or sypmtoms, preventive care appointment requests, appreciation or praise, and life issues did not converge.

Approved for Public Release; Distribution Unlimited. Public Release Case Number 20-3387 The author's affiliation with The MITRE Corporation is provided for identification purposes only and is not intended to convey or imply MITRE's concurrence with, or support for, the positions, opinions, or viewpoints expressed by the author. ©2020 The MITRE Corporation. ALL RIGHTS RESERVED

Table S4. Odds Ratios (95% Confidence Intervals) Estimating the Association between Clinic Staff Characteristics and Staff-Generated Message Content

Clinic staff characteristics	Action response				Information seeking	Deferred information sharing	Information sharing		Recommendation to schedule appointment
	Acknowledge	Fulfills request	Partially fulfills request	Denies request			Medical guidance	Orientation to procedures, treatments, or preventive behaviors	
**Staff type (vs Physician)**									
Administrative staff	0.38 (0.17-0.83)	2.01 (1.14-3.55)	0.21 (0.1-0.44)	1.64 (0.71-3.78)	0.75 (0.43-1.32)	0.52 (0.3-0.92)	0.04 (0.02-0.11)	0.63 (0.37-1.08)	0.12 (0.03-0.43)
Licensed practical nurse	1.83 (0.8-4.18)	0.63 (0.32-1.26)	1.28 (0.61-2.69)	0.59 (0.15-2.3)	0.97 (0.47-2.01)	1.47 (0.73-2.95)	0.85 (0.41-1.79)	0.84 (0.42-1.69)	1.08 (0.42-2.73)
Nurse Practitioner	2.21 (0.93-5.26)	0.78 (0.37-1.64)	3.21 (1.42-7.25)	2.18 (0.57-8.24)	1.49 (0.68-3.28)	1.17 (0.54-2.51)	2.74 (1.12-6.68)	0.94 (0.42-2.13)	2.18 (0.9-5.24)
Other clinician	0.47 (0.16-1.41)	1.36 (0.68-2.72)	0.63 (0.26-1.53)	0.4 (0.07-2.27)	0.54 (0.24-1.22)	0.7 (0.34-1.47)	1.42 (0.68-2.95)	1.72 (0.81-3.63)	1.08 (0.41-2.85)
Registered Nurse	1.38 (0.8-2.38)	0.97 (0.63-1.5)	1.36 (0.85-2.18)	1.34 (0.64-2.84)	1.14 (0.72-1.78)	1.61 (1.04-2.49)	1.4 (0.87-2.26)	1.66 (1.04-2.63)	1.44 (0.82-2.53)
**Clinical speciality (vs Primary care)**									
Not applicable	0.52 (0.27-1.01)	1.14 (0.68-1.9)	1.38 (0.78-2.47)	1.52 (0.58-4.02)	0.98 (0.57-1.68)	0.85 (0.5-1.45)	0.82 (0.46-1.43)	0.66 (0.38-1.14)	0.87 (0.47-1.59)
Specialist	1.32 (0.81-2.13)	0.88 (0.59-1.33)	0.8 (0.5-1.29)	0.93 (0.42-2.08)	0.81 (0.53-1.26)	1.09 (0.72-1.67)	0.9 (0.55-1.46)	1.23 (0.79-1.93)	1.08 (0.69-1.71)
**Number of messages sent in 2017 (vs >3400)**									
<=1000	0.41 (0.27-0.61)	0.46 (0.34-0.63)	0.47 (0.32-0.68)	0.54 (0.3-0.99)	0.41 (0.29-0.56)	0.51 (0.37-0.69)	0.3 (0.21-0.43)	0.48 (0.35-0.66)	0.51 (0.34-0.78)
1001-2000	0.71 (0.49-1.02)	0.78 (0.57-1.07)	0.56 (0.39-0.81)	0.72 (0.4-1.28)	0.45 (0.32-0.63)	0.53 (0.38-0.73)	0.81 (0.56-1.17)	0.67 (0.48-0.93)	0.67 (0.45-1.00)
2001-3400	1 (0.69-1.46)	1.1 (0.78-1.56)	1.35 (0.95-1.92)	0.82 (0.44-1.5)	1.66 (1.18-2.34)	1.12 (0.8-1.57)	1.57 (1.03-2.38)	1.24 (0.84-1.83)	1.33 (0.91-1.95)

Notes: Each column represents a separate logistic regression model for which the dependent variable is the column header and the rows represent the independent variables. Model for *Social communication/ Encouragement* did not converge.

Approved for Public Release; Distribution Unlimited. Public Release Case Number 20-3387 The author's affiliation with The MITRE Corporation is provided for identification purposes only and is not intended to convey or imply MITRE's concurrence with, or support for, the positions, opinions, or viewpoints expressed by the author. ©2020 The MITRE Corporation. ALL RIGHTS RESERVED

Table S5. Odds Ratios (95% Confidence Intervals) Estimating the Association between Patient Characteristics and Staff-Generated Message Content

Patient Characteristics	Action response			Information seeking	Deferred information sharing	Information sharing		Recommendation to schedule appointment
	Acknowledge	Fulfills request	Partially fulfills request			Medical guidance	Orientation to procedures, treatments, or preventive behaviors	
18-59 years vs 60+ years	0.83 (0.69-1.00)	1.15 (0.96-1.38)	0.76 (0.63-0.91)	0.97 (0.81-1.15)	1.05 (0.89-1.23)	0.84 (0.71-0.99)	1.14 (0.95-1.36)	1.02 (0.83-1.26)
Female vs Male	1.01 (0.84-1.21)	0.82 (0.69-0.98)	0.88 (0.74-1.05)	1.03 (0.87-1.22)	1.12 (0.95-1.31)	1.04 (0.89-1.22)	1.1 (0.92-1.31)	1.16 (0.94-1.44)
**Race (vs White)**								
Black	0.88 (0.62-1.26)	1.08 (0.79-1.49)	0.99 (0.72-1.35)	1.24 (0.91-1.69)	0.85 (0.64-1.14)	1.02 (0.76-1.37)	0.74 (0.53-1.03)	1.13 (0.79-1.61)
Other race	0.74 (0.41-1.31)	0.88 (0.53-1.46)	1.23 (0.74-2.03)	0.82 (0.5-1.35)	1.3 (0.82-2.06)	1.14 (0.72-1.81)	1.49 (0.87-2.54)	1.24 (0.71-2.18)
**Health condition (vs Both)**								
Diabetes	0.9 (0.71-1.15)	0.9 (0.71-1.14)	0.92 (0.73-1.17)	1.14 (0.91-1.44)	0.91 (0.74-1.13)	1.17 (0.94-1.45)	1.08 (0.85-1.37)	0.93 (0.71-1.22)
Hypertention	1.33 (1.04-1.69)	0.9 (0.71-1.13)	1.04 (0.82-1.32)	0.88 (0.71-1.1)	1.08 (0.88-1.33)	1.07 (0.87-1.33)	0.82 (0.65-1.03)	1.27 (0.97-1.68)
Non-urban vs Urban	1.2 (0.68-2.11)	0.54 (0.32-0.92)	1.26 (0.74-2.16)	1.01 (0.59-1.72)	1.19 (0.72-1.96)	1.22 (0.73-2.04)	1.23 (0.68-2.21)	0.49 (0.17-1.41)
**Payer Type (vs Private payer)**								
Other payer	1.15 (0.7-1.89)	1.18 (0.79-1.77)	0.95 (0.58-1.55)	1.53 (0.99-2.37)	0.83 (0.58-1.19)	1.24 (0.77-2.02)	1.12 (0.76-1.64)	1.21 (0.65-2.25)
Public payer	1.12 (0.69-1.81)	0.7 (0.48-1.04)	1.29 (0.8-2.06)	1.26 (0.83-1.92)	1.04 (0.73-1.46)	2.03 (1.26-3.25)	1.22 (0.84-1.78)	1.21 (0.66-2.21)
Uninsured	0.69 (0.2-2.42)	1.06 (0.42-2.69)	0.54 (0.16-1.86)	0.37 (0.13-1.06)	1.67 (0.73-3.81)	0.21 (0.06-0.73)	0.69 (0.29-1.63)	0.56 (0.11-2.86)
**Number of threads intiated (vs 8+)**								
1	0.25 (0.14-0.43)	0.14 (0.1-0.2)	0.13 (0.06-0.26)	0.14 (0.1-0.2)	0.27 (0.19-0.37)	0.25 (0.18-0.35)	0.18 (0.13-0.24)	0.16 (0.06-0.4)
2	0.5 (0.3-0.83)	0.46 (0.33-0.64)	0.51 (0.31-0.85)	0.56 (0.4-0.77)	0.49 (0.35-0.68)	0.47 (0.34-0.66)	0.43 (0.31-0.59)	0.85 (0.48-1.51)
3-4	0.71 (0.48-1.06)	1.07 (0.79-1.44)	1.35 (0.94-1.94)	0.87 (0.66-1.15)	0.88 (0.67-1.16)	0.91 (0.69-1.2)	0.94 (0.69-1.27)	0.68 (0.4-1.18)
5-7	1.93 (1.38-2.72)	1.57 (1.13-2.17)	1.92 (1.35-2.72)	1.83 (1.37-2.45)	1.70 (1.29-2.24)	1.46 (1.11-1.93)	1.72 (1.22-2.43)	1.95 (1.27-2.99)
**Distance to clinic in miles (vs 40+)**								
0-8	0.93 (0.68-1.25)	1.13 (0.84-1.53)	0.95 (0.71-1.28)	1.1 (0.83-1.47)	0.95 (0.73-1.23)	0.82 (0.63-1.08)	0.97 (0.72-1.31)	1.09 (0.79-1.51)
9-16	1.23 (0.92-1.63)	1.08 (0.81-1.44)	0.91 (0.68-1.21)	0.99 (0.75-1.3)	1.25 (0.97-1.62)	1.11 (0.86-1.44)	0.93 (0.7-1.24)	1.14 (0.82-1.59)
17-39	0.92 (0.68-1.24)	0.93 (0.71-1.22)	1.08 (0.82-1.43)	1.17 (0.9-1.52)	0.93 (0.72-1.19)	1.14 (0.89-1.47)	1.1 (0.84-1.45)	1 (0.72-1.39)
**Number of co-occurring conditions (vs 6-9)**								
1	0.83 (0.55-1.24)	0.92 (0.64-1.32)	0.79 (0.53-1.18)	1.24 (0.87-1.77)	1.17 (0.83-1.63)	1.26 (0.9-1.78)	1.2 (0.83-1.73)	1.03 (0.64-1.65)
2	0.98 (0.71-1.34)	0.96 (0.71-1.29)	0.85 (0.62-1.17)	0.82 (0.62-1.1)	0.93 (0.7-1.22)	1.06 (0.8-1.4)	1.06 (0.78-1.44)	0.68 (0.45-1.02)
3-5	0.85 (0.66-1.1)	0.95 (0.73-1.24)	1.08 (0.84-1.39)	0.92 (0.72-1.18)	0.93 (0.74-1.17)	0.9 (0.71-1.14)	0.89 (0.68-1.15)	1.29 (0.96-1.72)
**Number of outpatient visits (vs 21+)**								
1-5	0.9 (0.59-1.38)	1.16 (0.82-1.65)	0.99 (0.66-1.48)	0.97 (0.69-1.36)	0.77 (0.55-1.06)	0.86 (0.62-1.2)	0.78 (0.55-1.1)	0.92 (0.56-1.53)
6-10	0.86 (0.6-1.22)	1.01 (0.72-1.41)	1.04 (0.75-1.44)	1.19 (0.87-1.63)	1.11 (0.83-1.49)	1.14 (0.85-1.54)	1.1 (0.78-1.54)	0.99 (0.66-1.46)
11-15	0.85 (0.57-1.27)	1.55 (1.00-2.40)	1.26 (0.87-1.83)	0.78 (0.53-1.13)	0.89 (0.63-1.27)	1.03 (0.72-1.48)	1.44 (0.93-2.22)	1.12 (0.73-1.7)
16-20	1.45 (1.04-2.02)	0.88 (0.66-1.19)	0.89 (0.64-1.23)	0.93 (0.69-1.24)	1.05 (0.8-1.38)	0.9 (0.68-1.2)	0.75 (0.56-1.01)	1 (0.68-1.47)

Notes: Each column represents a separate logistic regression model for which the dependent variable is the column header and the rows represent the independent variables. Models for *request denials and Social communication/Encouragement* did not converge.

Approved for Public Release; Distribution Unlimited. Public Release Case Number 20-3387 The author's affiliation with The MITRE Corporation is provided for identification purposes only and is not intended to convey or imply MITRE's concurrence with, or support for, the positions, opinions, or viewpoints expressed by the author. ©2020 The MITRE Corporation. ALL RIGHTS RESERVED
